# Utilizing Zebrafish Embryos for Replication of Tulane Virus: A Human Norovirus Surrogate

**DOI:** 10.1007/s12560-024-09610-6

**Published:** 2024-08-23

**Authors:** Sahaana Chandran, Kristen E. Gibson

**Affiliations:** Department of Food Science, Center for Food Safety, University of Arkansas System Division of Agriculture, Fayetteville, AR 72704 USA

**Keywords:** Human norovirus, Tulane virus, Zebrafish larvae, Zebrafish embryo

## Abstract

The zebrafish larvae/embryo model has been shown to support the replication of seven strains (G1.7[P7], GII.2[P16], GII.3[P16], GII.4[P4], GII.4[P16], GII.6[P7], and GII.17[P13]) of human norovirus (HuNoV). However, due to challenges in consistently obtaining HuNoV-positive stool samples from clinical sources, evaluating HuNoV surrogates in this model is highly valuable. This study assesses the potential of zebrafish embryos and larvae as a model for Tulane virus (TuV) replication. Three infection methods were examined: microinjection, immersion, and feeding. Droplet digital PCR was used to quantify viral RNA across all three infection methods. Microinjection of 3 nL of TuV into zebrafish embryos (< 6-h post-fertilization) resulted in significant replication, with viral RNA levels reaching 6.22 logs at 4-day post-infection. In contrast, the immersion method showed no replication after immersing 4-day post-fertilization (dpf) larvae in TuV suspension for 6 h. Similarly, no replication was observed with the feeding method, where *Paramecium caudatum* loaded with TuV were fed to 4 dpf larvae. The findings indicate that the zebrafish embryo model supports TuV replication through the microinjection method, suggesting that TuV may serve as a useful surrogate for studying HuNoV pathogenesis. Additionally, TuV can be utilized in place of HuNoV in method optimization studies using the zebrafish embryo model, circumventing the limited availability of HuNoV.

## Introduction

Human norovirus (HuNoV) is the leading cause of non-bacterial acute gastroenteritis worldwide (Ahmed et al., 2014; Cannon et al., 2021; Liao et al., 2021), responsible for a significant burden of foodborne illnesses, with approximately 125 million cases annually (Kirk et al., 2015). The economic impact of HuNoV is substantial, incurring an estimated $4.2 billion in direct health system costs and $60.3 billion in societal costs globally each year (Bartsch et al., 2016). Despite its prevalence, understanding HuNoV pathogenesis has been challenging due to difficulties in culturing the virus in vitro, related to its enteric nature and the incomplete knowledge of cell surface entry receptor proteins that facilitate internalization. Efforts to develop a facile cell culture system for HuNoV replication have largely been unsuccessful (Duizer et al., 2004). Consequently, surrogate viruses such as feline calicivirus (FCV), murine norovirus (MNV), and Tulane virus (TuV) are frequently used in HuNoV research for studies on inactivation, disinfection, environmental stability, and persistence, as well as to predict HuNoV transmission dynamics for risk modeling purposes (Cannon et al., 2006; Cromeans et al., 2014; Hirneisen & Kniel, 2013; Kamarasu et al., 2018; Sattar et al., 2011; Tung-Thompson et al., 2015).

Historically, HuNoV detection in food, water, and environmental samples has relied on molecular detection methods. However, detecting viral RNA does not necessarily indicate the presence of infectious virus, thus necessitating infectivity assays (Chandran & Gibson, 2024). Recently, a human intestinal enteroid (HIE) model capable of supporting the replication of multiple HuNoV strains was described (Ettayebi et al., 2016). Since its development, the HIE model has been utilized in various studies investigating norovirus persistence (Desdouits et al., 2022; Kennedy et al., 2023; Shaffer et al., 2022), inactivation (Hayashi et al., 2021), and quantification in soft berries (Wales et al., 2023). However, the HIE model is labor intensive and costly due to the various growth factors and medium required for culturing the cells (Chandran & Gibson, 2024). Another recent robust small animal model involves using zebrafish larvae and embryos, which support the replication of HuNoV strains from both GI and GII genogroups (Tan et al., 2022, 2023; Van Dycke et al., 2019). Zebrafish share orthologues for almost 70% of human genes and their gastrointestinal tract development and physiology are similar to humans (Howe et al., 2013; Lickwar et al., 2017). Additionally, the zebrafish model is more cost-effective compared to the HIE model.

In this study, TuV was chosen to investigate whether it replicates in zebrafish embryo/larva, analogous to that observed with select HuNoV strains. The significance of TuV replication in zebrafish embryos/larvae lies in its relevance as a surrogate for HuNoV. Tulane virus, belonging to the *Caliciviridae* family, binds to histo-blood group antigens as attachment factors for infection like HuNoV (Farkas et al., 2010; Zhang et al., 2015). Furthermore, recent findings have demonstrated that TuV relies on intracellular calcium for replication, with the NS1-2 protein functioning as a viroporin to alter cellular calcium signaling, thereby facilitating TuV replication. Norovirus NS1-2 protein induces similar changes in cellular calcium signaling, suggesting a shared physiological function between HuNoV and TuV (Strtak et al., 2019). Additionally, previous research has shown that TuV exhibits environmental persistence, disinfection, and physicochemical properties comparable to those of HuNoV (Arthur & Gibson, 2015; Drouaz et al., 2015; Faircloth et al., 2022; Stoppel et al., 2023). Therefore, demonstrating TuV replication in the zebrafish embryo/larva model, as observed with HuNoV, would suggest that TuV and HuNoV share similar pathogenesis mechanisms.

Human norovirus transmission occurs through direct person-to-person contact, ingestion of contaminated food and water, or via environmental fomites. The second most common transmission route for HuNoV, after direct person-to-person contact, is through contaminated food and water (de Graaf et al., 2016; Lopman et al., 2012). To mimic these infection routes observed in humans, this study considered both immersion and a novel feeding method alongside the microinjection method for infecting zebrafish larvae. Van Dycke et al. (2019) investigated immersion and microinjection methods for infecting zebrafish larvae with HuNoV strains, finding replication only with the microinjection method, not immersion. Similarly, Tan et al. (2022, 2023) observed HuNoV replication in both larvae and embryos via microinjection. To verify that immersion is not a viable infection route, the immersion method was included in this study along with microinjection and a novel feeding method.

## Materials and Methods

### Zebrafish Husbandry and Maintenance

Wildtype (AB strain) adult zebrafish were purchased from Zebrafish International Resource Center (ZIRC, Eugene, OR) and were housed in a standalone tabletop rack re-circulatory aquaculture system (eRack) equipped with an ultraviolet sterilizer, carbon filters, and a fluidized bed biological filter (Aquaneering, Inc., San Marcos, CA). The water temperature was maintained at 28 °C and a 14-h light/10-h dark cycle was implemented. Fertilized eggs were collected from adults placed in mating cages and kept in petri dishes containing Danieau’s solution (1.5-mM HEPES, 17.4-mM NaCl, 0.21-mM KCl, 0.12-mM MgSO_4_, and 0.18-mM Ca(NO_3_)_2_, and 0.6-μM methylene blue). All zebrafish experiments were performed in compliance with the Institutional Animal Care and Use Committee guidelines, University of Arkansas, Fayetteville (AUP22020, AUP23012).

### Tulane Virus Production and Quantification

Tulane virus was provided by Dr. Jason Jiang (Cincinnati Children’s Hospital Medical Center, Cincinnati, OH) and propagated in monkey kidney cells LLC-MK2 (ATCC CCL-7; Manassas, VA). The production, storage, and titration of TuV stocks were done following the method described by Arthur and Gibson (2015) with slight modifications. After centrifuging at 3000×*g* for 15 min at 4 °C, the harvested TuV supernatant was filtered through a 0.45-µm filter pore bottle top vacuum filter (Corning Inc., Corning, NY) that had been pre-treated with 1% Tween 80, followed by washing the filter with 1× phosphate-buffered saline (PBS). The filtered supernatant was then aliquoted into 1-mL cryovials and stored at − 80 °C.

### Microinjection of Tulane Virus into Zebrafish Embryos

For virus microinjection, fertilized eggs were collected and washed three times with 0.3 × Danieau’s solution. The embryos were then transferred to a petri dish with grooves created by a mold imprint (6 rows of V-shaped grooves) in 1.5% agarose. Injection needles were pulled out of glass capillaries using a micropipette puller with a heat filament (Sutter Instruments, Novato, California). Microinjection was done using a M3301R Manual Micromanipulator (WPI) and a Nanoject III Microinjector (Drummond Scientific Company, Broomall, PA). Each zebrafish embryo was injected within 6-h post-fertilization (hpf) with 3 nL of TuV, while the negative control embryos were injected with 3 nL of 1× PBS. After injection, zebrafish embryos were transferred to 6-well plates with Danieau’s solution and maintained in an incubator with a 14/10 h light/dark cycle at 29.5 °C. The embryos were observed daily post-injection to ensure proper development, with any dead embryos or larvae being removed and the medium being changed daily. Ten zebrafish embryos/larvae were pooled as one sample, and 2 samples were collected for 5-day post-infection (dpi) into 2-mL tubes and stored at − 80 °C.

### Immersion of Zebrafish Larvae in Tulane Virus

Four dpf larvae were immersed in TuV suspension (~ 1 × 10^7^ PFU/mL) diluted in Danieau’s solution for 6 h. After exposure, the larvae were washed three times in Danieau’s solution and transferred to 6-well plates having new medium. The larvae were incubated in an incubator with a 14/10-h light/dark cycle at 29.5 °C. The larvae were monitored daily post-infection for posture and swimming behavior. Ten zebrafish larvae were pooled as one sample, and 2 samples were collected for 5 dpi into 2-mL tubes and stored at − 80 °C.

### *Paramecium caudatum* Culturing

*P. caudatum* culture was purchased from ZIRC and were cultured according to ZIRC Paramecia SOP 2016_1 (ZIRC, 2016), with slight modifications. Briefly, 10 mL of starter *P. caudatum* culture was added to 100 mL of reverse osmosis (RO) water in a 175 cm^2^ tissue culture flask along with 0.01 g of powdered brewer’s yeast and 5 wheat berries which were previously autoclaved and boiled for 10 min in RO water. The paramecia cultures were maintained at 26 °C for 4 weeks before starting new cultures.

### Incubation of *Paramecium caudatum* with Tulane Virus

Five autoclaved wheat berries were boiled for 10 min in RO water. Later, the water was drained, and the wheat berries were allowed to cool down. The wheat berries were added to a 25-cm^2^ tissue culture flask along with 5 mL of TuV (10^6^ PFU/mL). The flask was incubated at room temperature with gentle rocking overnight to allow attachment of TuV to the wheat berries. Next day, 5 mL of paramecia (10^5^ cells/mL), which were given limited food (2 wheat berries instead of 5) the previous day, were added to the flask and incubated at room temperature with gentle rocking for 2 h. After incubation, the paramecia-virus suspension was transferred to a 15-mL centrifuge tube leaving out the wheat berries. The suspension was centrifuged for 5 min at 300×*g* to pellet the paramecia cells. The supernatant was discarded, and the pellet was washed with 5 mL 1× PBS to remove any unassociated virus. This step was repeated 3× times and then the paramecia cells were suspended in 5-mL PBS.

### Feeding of Tulane Virus to Zebrafish Larvae

Five mL of TuV-associated *P. caudatum* was fed to 4 dpf larvae kept in a petri dish (100 × 15 mm) having Danieau’s medium. After 2 h of feeding, the larvae were washed with fresh Danieau’s medium 3×, and 10 larvae per sample were collected in 2-mL tubes for 5-day post-feeding TuV-associated *P. caudatum* and then stored at − 80 °C.

### RNA Extraction from Zebrafish Embryos and Larvae

Zebrafish larvae, harvested and stored at − 80 °C in 2-mL tubes, were mixed with 700 μL of TRI reagent (Zymo Research, Irvine, CA) and then homogenized using a handheld VWR 200 Homogenizer Unit fitted with a 5 × 75 mm flat-bottom generator probe (Cat No: 10032-324, VWR International, LLC). The homogenizer was set to position 1, corresponding to a speed range of 5000–8000 rpm. The larvae samples were homogenized for 3 cycles of 15 s, with rest intervals of 60 s. The homogenates were clarified by centrifugation at 9000×*g* for 5 min, and RNA was extracted using the Direct-zol RNA Miniprep (Zymo Research, Irvine, CA) following the manufacturer’s protocol.

### Droplet Digital PCR for Quantification of Viral RNA Copies

To detect viral RNA copies, droplet digital PCR (ddPCR) was used. One-Step RT-ddPCR Advanced Kit for Probes (Bio-Rad Laboratories, Hercules, CA) was used along with 900 nM of each primer, 250 nM of the probe, and 15-nM dithiothreitol. The specific primers and probes for TuV detection were as follows: TuV forward (5’-TGACGATGACCTTGCGTG-3’), TuV reverse (5’-TGGGATTCAACCATGATACAGTC-3’), and TuV probe (5HEX/ACCCCAAAG/ZEN/CCCCAGAGTTGAT/3IABkFQ) (Integrated DNA Technologies, Inc., San Diego, CA) (Tian et al., 2013). Each 20-μL sample was loaded into DG8™ Cartridges (Bio-Rad Laboratories), followed by the addition of 70 μL of Droplet Generation Oil for Probes (Bio-Rad Laboratories). Droplets were generated using the QX200™ Droplet Generator (Bio-Rad Laboratories). The droplet suspension was then carefully transferred into 96-well plates by pipetting. The plates were sealed with the PX1 PCR Plate Sealer (Bio-Rad Laboratories) before conducting PCR in a C1000 Touch™ Thermal Cycler (Bio-Rad Laboratories). The thermal cycling program included a reverse transcription reaction at 47 °C for 60 min, an enzyme activation step at 95 °C for 10 min, followed by 40 PCR cycles of 95 °C for 30 s (denaturation, ramp rate 3 °C/s) and 53 °C for 1 min (annealing/extension, ramp rate 3 °C/s). The final step involved DNA polymerase deactivation at 98 °C for 10 min. Plates were held at 4 °C overnight before being analyzed using the QX200™ Droplet Digital™ PCR system (Bio-Rad Laboratories). Results were visualized and analyzed using QuantaSoft™ version 1.7.4, with thresholds for distinguishing positive and negative clusters manually set above the negative cluster.

### Statistical Analysis

Three experimental trials were conducted for each infection route, with two samples collected per trial. The data were analyzed using a linear model, as they satisfied the assumptions of normality and heteroscedasticity. Treatment means and their 95% confidence intervals were calculated using estimated marginal means. Multiple pairwise comparisons were performed to identify statistical differences at *P* = 0.05. The analysis was carried out in R Studio (R Core Team, 2022) using the base, ggplot2 (Wickham, 2016), ggpubr (Kassambara, 2020), and emmeans (Length et al., 2021), multcomp (Hothorn et al., 2008), and multcompView (Graves et al., 2019) packages.

## Results

### Microinjection of Zebrafish Embryos with Tulane Virus

**Fig. 1:**
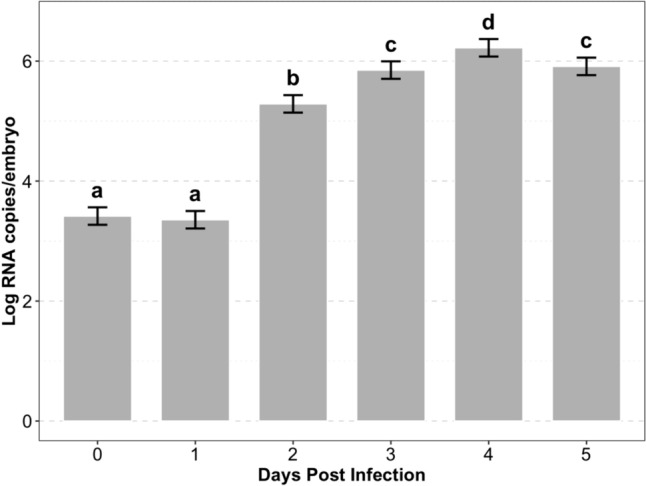
Droplet digital PCR quantification of TuV RNA genome copies/embryo obtained from analysis of variance with multiple pairwise comparisons for microinjection method from 0 to 5 dpi. The error bars represent 95% confidence intervals and compact letter format is used to designate statistical difference between days at *P* = 0.05

The estimated log RNA copies per embryo each day after microinjection of TuV into zebrafish embryos are plotted in Fig. 1. A significant increase in TuV RNA copies, exceeding 5 logs per embryo, was observed at 2 dpi. The highest levels of the virus were detected at 4 dpi (6.22 log RNA copies/embryo). On the last day tested (5 dpi) the viral RNA levels declined to below 6 logs, similar to the levels observed at 3 dpi.

### Immersion of Zebrafish Larvae with Tulane Virus

**Fig. 2:**
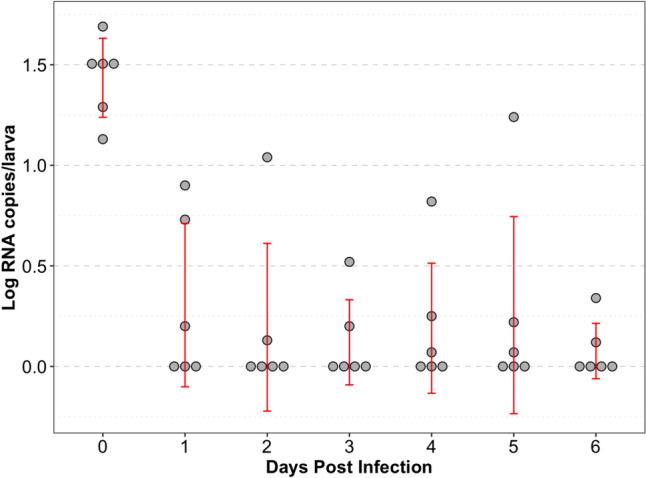
Raw data values of TuV RNA genome copies/larva obtained by droplet digital PCR quantification for immersion method from 0 to 6 dpi

The raw data of log RNA copies per larva for each day following the immersion of 4 dpf larvae in TuV suspension are presented in Fig. 2. No replication of TuV was observed in the zebrafish larvae post-immersion.

### Feeding of Zebrafish Larvae with Tulane Virus

**Fig. 3:**
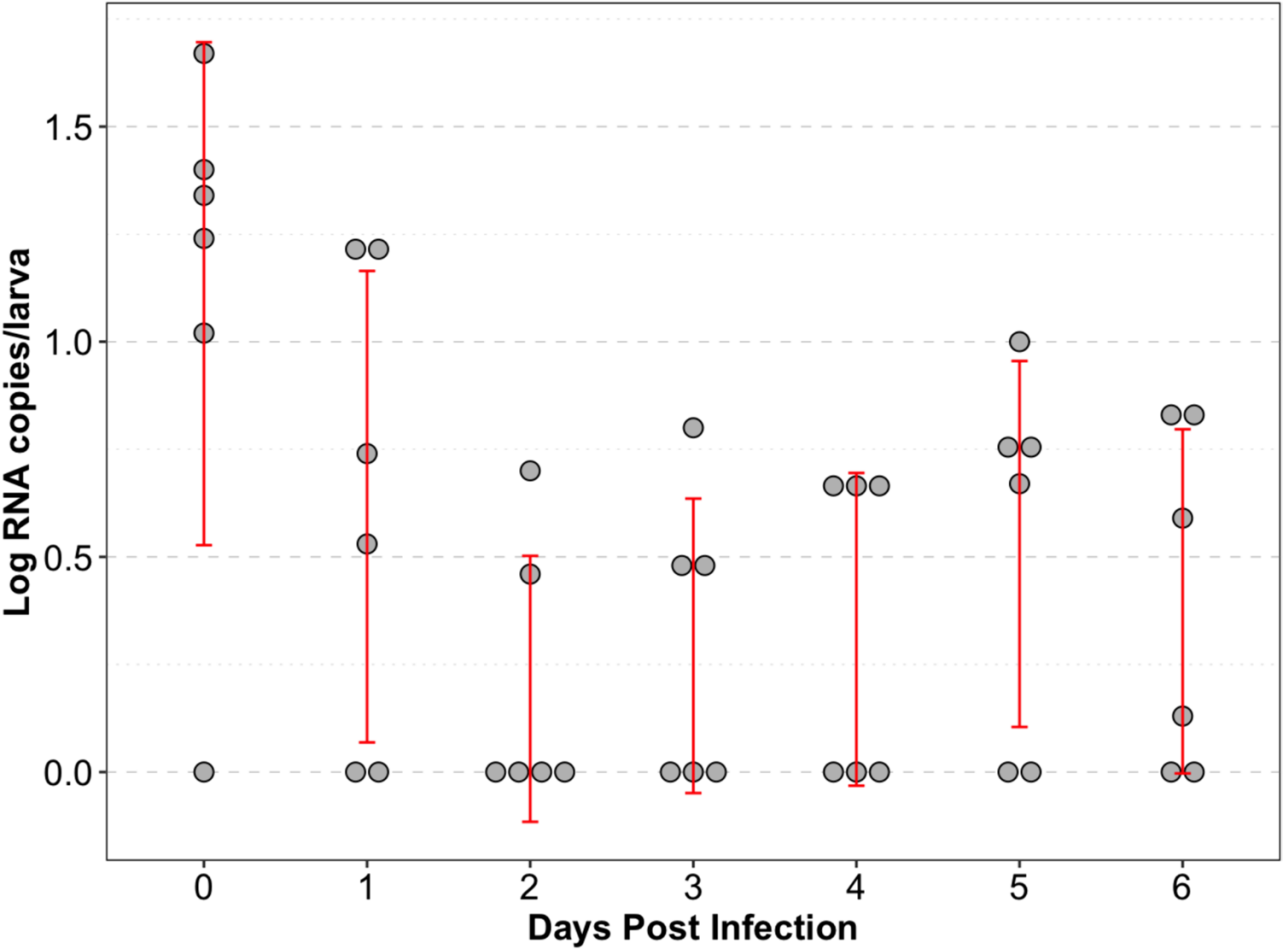
Raw data values of TuV RNA genome copies/larva obtained by droplet digital PCR quantification for feeding method from 0 to 6 dpi

When paramecia were fed wheat berries associated with TuV, viral ingestion was confirmed via a plaque assay. Briefly, paramecia samples (500 μL) were collected at 0, 1, 2, and 3 h after incubation with TuV. At 0 h, 2.6 PFU of TuV was present in each paramecium. The amount of TuV present in each paramecium decreased over time, with 1.06 PFU at 1 h, 1.02 PFU at 2 h, and 0.84 PFU at 3 h. Figure 3 shows the raw values of log RNA copies per larva per day after 4 dpf larvae were fed TuV-loaded *P. caudatum*. No TuV replication was detected in the zebrafish larvae following this feeding.

## Discussion

In this study, TuV, a surrogate for HuNoV, was tested for its replication using the zebrafish embryo model through microinjection. Replication of select strains of HuNoV has been reported in both zebrafish larvae and embryos using the microinjection method (Tan et al., 2023; Van Dycke et al., 2019). However, the zebrafish embryo model was chosen over the zebrafish larvae model because microinjection into zebrafish embryos resulted in greater replication of HuNoV compared to zebrafish larvae, and embryo microinjection is easier and more time efficient than larvae microinjection (Tan et al., 2023). Tan et al. (2023) reported that microinjection of select GII strains of HuNoV (GII.4[P16], GII.2[P16], and GII.17[P13]) into zebrafish embryos led to an increase in viral RNA levels of over 6 logs/embryo at 2 dpi. With TuV microinjection in the present study, viral RNA levels of over 5 logs/embryo were observed at 2 dpi. The peak viral RNA replication with TuV microinjection was observed on day 4, with 6.22 log viral RNA/embryo. Meanwhile, with HuNoV microinjection, the peak viral RNA detection levels were reached at 2 dpi (over 6 logs genome copies/embryo) (Tan et al., 2023). On 5 dpi, the viral RNA levels of both HuNoV strains as well as TuV had reduced to comparable levels of below 6 log genome copies/embryo.

In the zebrafish larvae model, the expression of terminal fucoses as part of histo-blood group antigens (HBGAs) has been shown to be a requirement for the successful replication of HuNoV (Cuvry et al., 2022). Histo-blood group antigens have been identified as one of the receptors required for infection for certain strains of HuNoV based on in vitro binding assays (Huang et al., 2005; Hutson et al., 2003; Marionneau et al., 2002) and human volunteer challenge studies (Hutson et al., 2002; Lindesmith et al., 2003). Similarly, HBGAs have been shown to serve as attachment factors for TuV as well, through in vitro studies (Farkas et al., 2010; Zhang et al., 2015). The similar replication trend of TuV in the same zebrafish embryo model that supported the replication of three different GII strains of HuNoV can be attributed to the fact that TuV also binds to the same attachment factors, i.e., HBGAs, as some strains of HuNoV. Conversely, MNV, another widely used surrogate for HuNoV, did not replicate when microinjected into zebrafish larvae at 3 dpf (Van Dycke et al., 2019). Murine norovirus has been shown to directly interact with two murine cell surface molecules, CD300lf and CD300ld, for successful replication in RAW264.7 cells (Haga et al., 2016). These molecules might not be encoded by zebrafish, which could explain the lack of MNV replication in the zebrafish larvae model (Van Dycke et al., 2019).

The second inoculation method tested in this study was the immersion of 4 dpf zebrafish larvae in TuV suspension. As immersion might represent a more natural infection route along with previous research on immersion of 5 dpf and 28 dpf zebrafish larvae in rotavirus suspension showing significant increase in viral replication (Song et al., 2022), this method was considered. However, no replication of TuV was observed after 6 h of immersion. Similar results were observed when 4–5 dpf zebrafish larvae were immersed in GII.4[P4] for 8 h (Van Dycke et al., 2019). Although both HuNoV and rotavirus are intestinal RNA viruses, differences in their viral structural proteins and pathogenesis mechanisms likely impacted the effectiveness of the immersion infection route. Based on the strain of rotavirus used by Song et al. (2022), the viral protein (VP) 8 domain of VP4 interacts with different glycans present on the surface of the host cell. For many human rotavirus strains, VP8 has been shown to bind to HBGAs; however, some strains of rotavirus that are detected in both animals and humans have been shown to bind to gangliosides as well (Ramani et al., 2016). These differences likely impact the contrasting success of the immersion route of infection between TuV, HuNoV, and rotavirus.

Foodborne transmission of HuNoV has been identified as a significant factor in the global dissemination of various HuNoV strains (de Graaf et al., 2016). To mimic this infection pathway, we investigated a novel feeding method employing *Paramecium caudatum*, a free-living, ciliated protists, and natural food source for zebrafish larvae from 4 dpf (Flores et al., 2019), to deliver TuV to zebrafish larvae. Previous studies have demonstrated that *Escherichia coli* and *Salmonella Typhimurium* ingested by *P. caudatum* led to bacterial colonization in the gastrointestinal tract when fed to the zebrafish larvae (Fan et al., 2019; Flores et al., 2019; Stones et al., 2017). Additionally, surrogates for HuNoV, namely MNV and FCV, have been shown to persist within two species of free-living amoebae (*Acanthamoeba castellanii* and *A. polyphaga*) for up to 8 days (Hsueh & Gibson, 2015). Other human pathogenic viruses such as adenovirus, coxsackievirus, rotavirus, and reovirus have also been shown to be ingested by free-living amoeba (Alotaibi, 2011; Folkins et al., 2020; Lorenzo-Morales et al., 2007; Mattana et al., 2006; Scheid & Schwarzenberger, 2012; Staggemeier et al., 2016; Verani et al., 2016.) In our study, TuV persisted within *P. caudatum* for at least 3 h. However, feeding TuV-loaded *P. caudatum* to zebrafish larvae did not result in viral replication. This outcome may be attributed to insufficient TuV concentration delivered by *P. caudatum* to each larva. Future research should explore the effects of multiple feedings with TuV-loaded *P. caudatum* to determine if this approach can achieve successful viral replication.

Currently, the only source of obtaining HuNoV is from human stool samples obtained from natural infections or human volunteer trials and not all strains are readily available in sufficient quantities for all research activities. For effective infection in the zebrafish larvae/embryo model, a high concentration of HuNoV titer (at least 8 log genome copies/g of stool) is required (Tan et al., 2022); however, not all norovirus-positive stool samples meet this concentration. Although this constraint can be overcome by employing virus concentration methods, some stool samples may contain substances that are toxic to zebrafish embryos/larvae and result in their death upon microinjection. This toxicity was observed in our lab, where microinjection of undiluted stool samples led to embryo mortality within an hour (data not shown). Therefore, some stool samples need to be diluted to reduce toxin concentration, which might also reduce the concentration of HuNoV present. This is also a challenge when using the HIE infectivity model. Wales et al. (2023) reported that human norovirus-positive stool samples used for infecting HIEs often required a five-fold dilution of a 10% stool suspension in PBS to avoid cytotoxicity. In some cases, even after dilution, stool samples may still be toxic to zebrafish when microinjected, rendering the stool sample unusable. Hence, to further understand and robustly utilize the zebrafish larvae/embryo model for HuNoV research, the model can be optimized using TuV.

In summary, TuV replication was supported only through the microinjection method in the zebrafish embryo model. Unfortunately, both immersion and feeding routes of infection did not result in successful replication of TuV in zebrafish larvae. However, the replication of TuV in the zebrafish embryo model, which has also supported the replication of certain strains of HuNoV, suggests the involvement of similar pathogenesis mechanisms, thus providing significant insights. As the zebrafish larvae and embryo models are relatively new, optimization of the zebrafish embryo model is necessary for the effective detection of HuNoVs from food, water, and environmental fomite samples, which may contain toxic and inhibitory substances, such as lipids, carbohydrates, and bacteria. Tulane virus can potentially be used in place of HuNoV in such optimization studies.

## Data Availability

Data are provided within the manuscript or supplementary information files.
